# Monitoring and analysis of surface deformation in alpine valley areas based on multidimensional InSAR technology

**DOI:** 10.1038/s41598-023-39677-3

**Published:** 2023-08-09

**Authors:** Fan Yang, Yan An, Chuang Ren, Jia Xu, Jinbo Li, Dongliang Li, Zhiwei Peng

**Affiliations:** 1 Institute of Science and Technology, Liaoning Technical University, Fuxin, 123000 China; 2 School of Geomatics, Liaoning Technical University, Fuxin, 123000 China; 3 Shanxi Changping Coal Industry Co., LTD., Jincheng, 046700 China; 4 Jinneng Holding Equipment Manufacturing Group, Zhaozhuang Coal Industry Co., LTD., Changzhi, 046600 China

**Keywords:** Natural hazards, Engineering

## Abstract

Joshimath has received much attention for its massive ground subsidence at the beginning of the year. Rapid urbanization and its unique geographical location may have been one of the factors contributing to the occurrence of this geological disaster. In high mountain valley areas, the complex occurrence mechanism and diverse disaster patterns of geological hazards highlight the inadequacy of manual monitoring. To address this problem, the inversion of deformation of the Joshimath surface in multiple directions can be achieved by multidimensional InSAR techniques. Therefore, in this paper, the multidimensional SBAS-InSAR technique was used to process the lift-track Sentinel-1 data from 2020 to 2023 to obtain the two-dimensional vertical and horizontal deformation rates and time series characteristics of the Joshimath ground surface. To discover the causes of deformation and its correlation with anthropogenic activities and natural disasters by analyzing the spatial and temporal evolution of surface deformation. The results show that the area with the largest cumulative deformation is located in the northeastern part of the town, with a maximum cumulative subsidence of 271.2 mm and a cumulative horizontal movement of 336.5 mm. The spatial distribution of surface deformation is based on the lower part of the hill and develops towards the upper part of the hill, showing a trend of expansion from the bottom to the top. The temporal evolution is divided into two phases: gentle to rapid, and it is tentatively concluded that the decisive factor that caused the significant change in the rate of surface deformation and the early onset of the geological subsidence hazard was triggered by the 4.7 magnitude earthquake that struck near the town on 11 September 2021.

## Introduction

Ground subsidence is a phenomenon in which the earth's surface continues to sink over time and produces hazards. It is a process of rock formation in which the loose layers of the earth's surface change from loose to fine under the action of gravity due to natural disasters or anthropogenic activities^1–3^. With the rapid development of urbanization in the world today, the development and construction of groundwater, minerals, and other resources and infrastructure have further accelerated the rate of surface subsidence, making ground settlement one of the major geological hazards facing mankind^4–6^. Large-scale ground subsidence not only destroys natural ecosystems but also has a serious impact on the development of the economy and society and the outcome of people's daily production and life.

Joshimath is an important frontier town in northern India, that combines economy, culture, and strategy. According to news reports, in January 2023, the town suffered from extensive ground subsidence, with all nine urban areas of Joshimath spared, resulting in irreversible structures such as cracks in the ground and tilting and collapsing houses. The impact of natural disasters such as earthquakes, landslides, mudslides, floods, extreme rainfall, etc.^7–10^ and the uncontrolled and massive construction of infrastructure by the government have overwhelmed the foundations of the town and indirectly created this serious geological disaster. The town of Auli, an important strategic base for the Indian Army, sits on the slopes directly above Joshimath. Is the subsidence still increasing in extent? This is an unprecedented challenge to the productive lives of the town's inhabitants and the Indian military. Therefore, the long-term monitoring of surface deformation in the city of Joshimath and the analysis of the spatial distribution and spatial and temporal evolution of the deformation can provide an effective scientific basis for further prevention and mitigation work and is also important for understanding the mechanism of the occurrence of subsidence hazards in the city of Joshimath and building a theoretical system for disaster prevention and control.

Interferometric synthetic aperture radar (InSAR) technology has developed rapidly in recent years, and its all-weather, wide-area, high-accuracy characteristics have enormous advantages over conventional surface monitoring methods^11,12^. Synthetic aperture radar (SAR) can acquire high-density sampling points covering the same area continuously, and generate high-resolution deformation rate fields after the InSAR data is decomposed^13^. InSAR technology has been widely used by experts and scholars in the field of geological hazard identification and detection, such as earthquakes, landslides, mines, and regional ground subsidence, and is becoming an indispensable new modern measurement technology^14–17^.

The small baseline subset (SBAS) technique is a development of the traditional D-InSAR technique, which is designed to extract surface deformation information at low resolution and large scale. It can effectively overcome errors such as atmospheric effects, reduce the influence of spatial and temporal coherence, improve the temporal sampling rate and spatial density of the observation data, and can obtain the surface deformation characteristics over a continuous period to analyze the deformation information and evolution patterns in the study area^18–20^. Ding et al. conducted a time-series monitoring of Baiyun District, Guangzhou City, using SBAS-InSAR technology and analyzed the causes of deformation with optical images. The results demonstrate that the deformation is mainly related to the local geological conditions and human activities (e.g., construction of buildings and structures)^21^. Wu et al. analyzed the correlation between urban surface deformation and urban earthworks construction in downtown Yan'an and the Yan'an new Airport (YNA) using the SBAS-InSAR technique. The present work shows that the subsidence originates from the earth's filling and a load of urban buildings, while the release of stress is the major factor for the land uplift. Moreover, it is found that the collapsibility of loess and concentrated precipitation deteriorates the degree of local land subsidence^22^. The above studies can provide a reference for ground settlement monitoring and causation analysis in urban areas. However, due to the characteristics of SAR side-view imaging, the SBAS-InSAR technique based on single-track data can only capture the projection of the real surface deformation in the radar line of sight (LOS) direction. For regional subsidence structures with both vertical deformation and horizontal movement, the results generated by the conventional SBAS-InSAR technique are highly inaccurate and do not represent the true surface deformation^23^. The Multidimensional SBAS-InSAR (MSBAS-InSAR) technique was developed to compensate for the shortcomings of the single-track InSAR technique, which can only capture LOS deformation. The method is based on SAR images with different orbits and different incidence angles to solve the time-series InSAR deformation values and use them as constraints for joint inversion to obtain the true vertical and horizontal two-dimensional deformation results of the surface^24^. Shaochun Dong and Yaoyao Ren et al. experimentally demonstrated the feasibility of MSBAS-InSAR technology in the field of deformation monitoring, providing a more comprehensive means of monitoring regional surface deformation^25,26^.

The Joshimath urban area is built in a complex alpine valley area and the unique geological conditions and rapid urbanization have led to significant ground subsidence in the Joshimath urban area. As an emergency measure, delineating the critical area boundaries and conducting a reasonable characterization should be the town's current priority. Therefore, this paper uses MSBAS-InSAR technology to monitor the surface deformation of the Joshimath urban area for three years based on the ascending and descending orbital Sentinel-1A data. The spatial distribution of deformation is used to analyze its spatial and temporal evolution, explore the correlation with natural disasters and anthropogenic activities, and discuss the future trends of surface deformation in the town. The study further provides a sound scientific basis for the economic development and disaster prevention and control of the Joshimath urban area.

## Description of the research area

**Figure 1:**
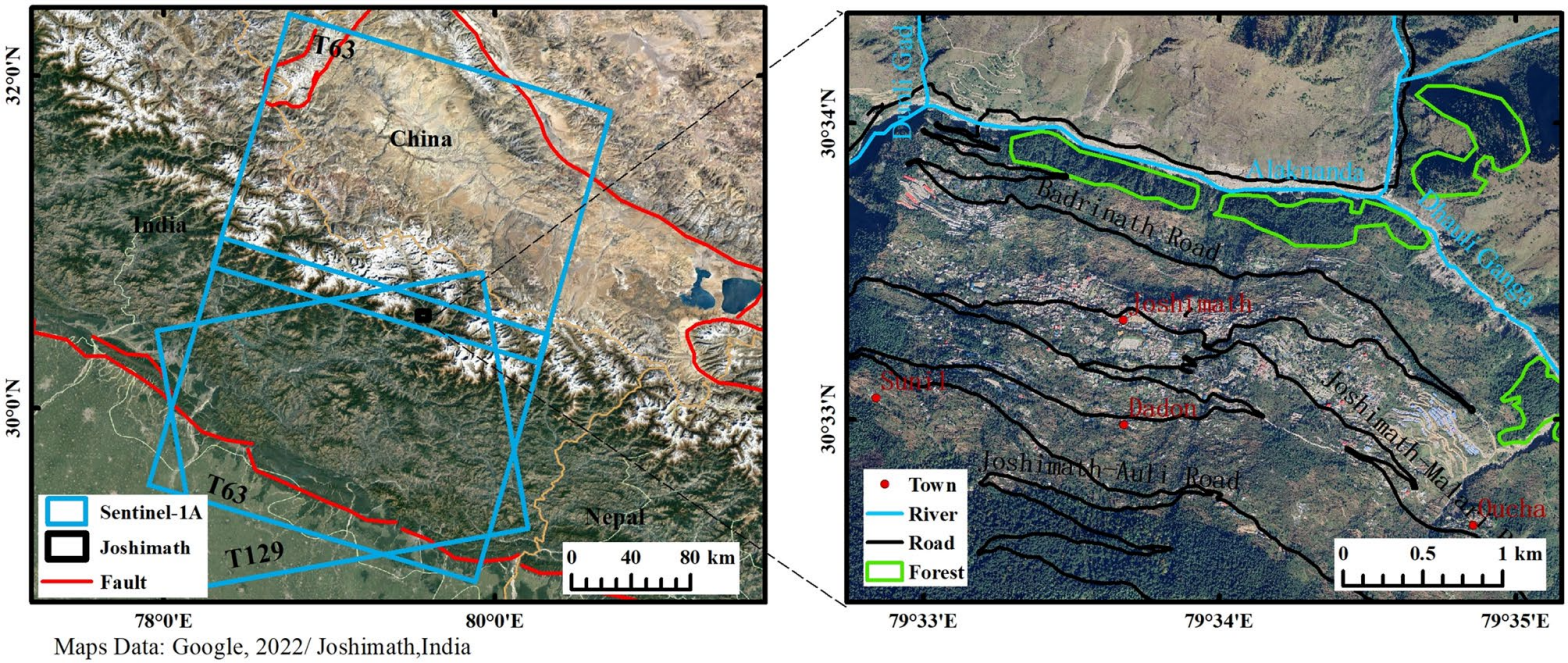
Geographical location and remote sensing images of Joshimath.

**Figure 2:**
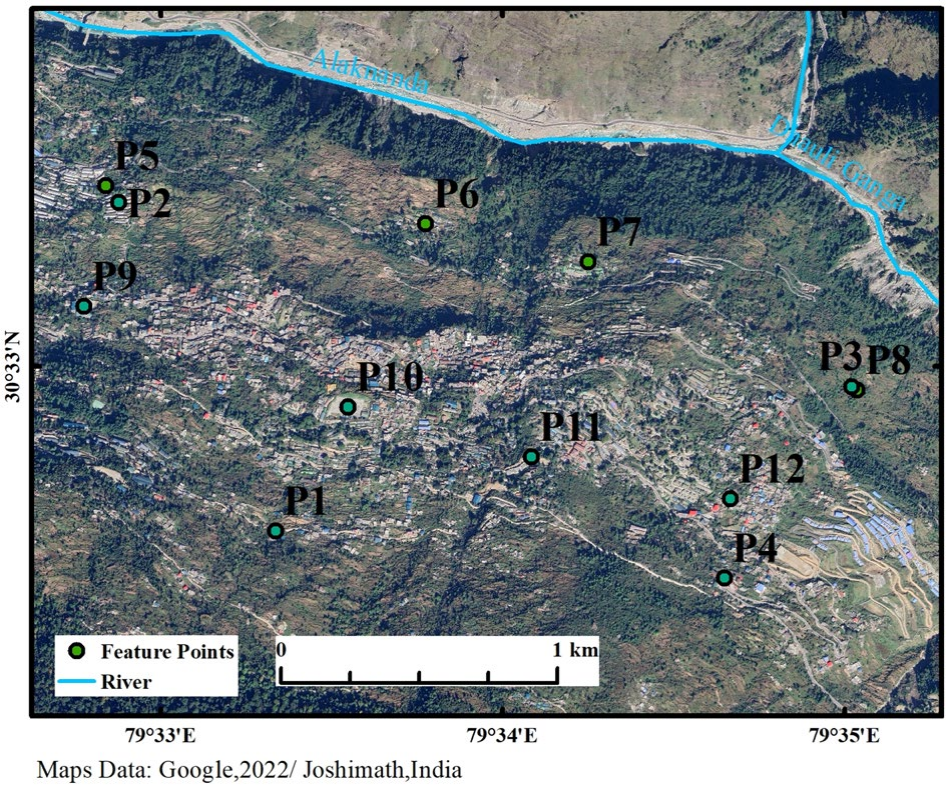
Feature point distribution.

**Figure 3:**
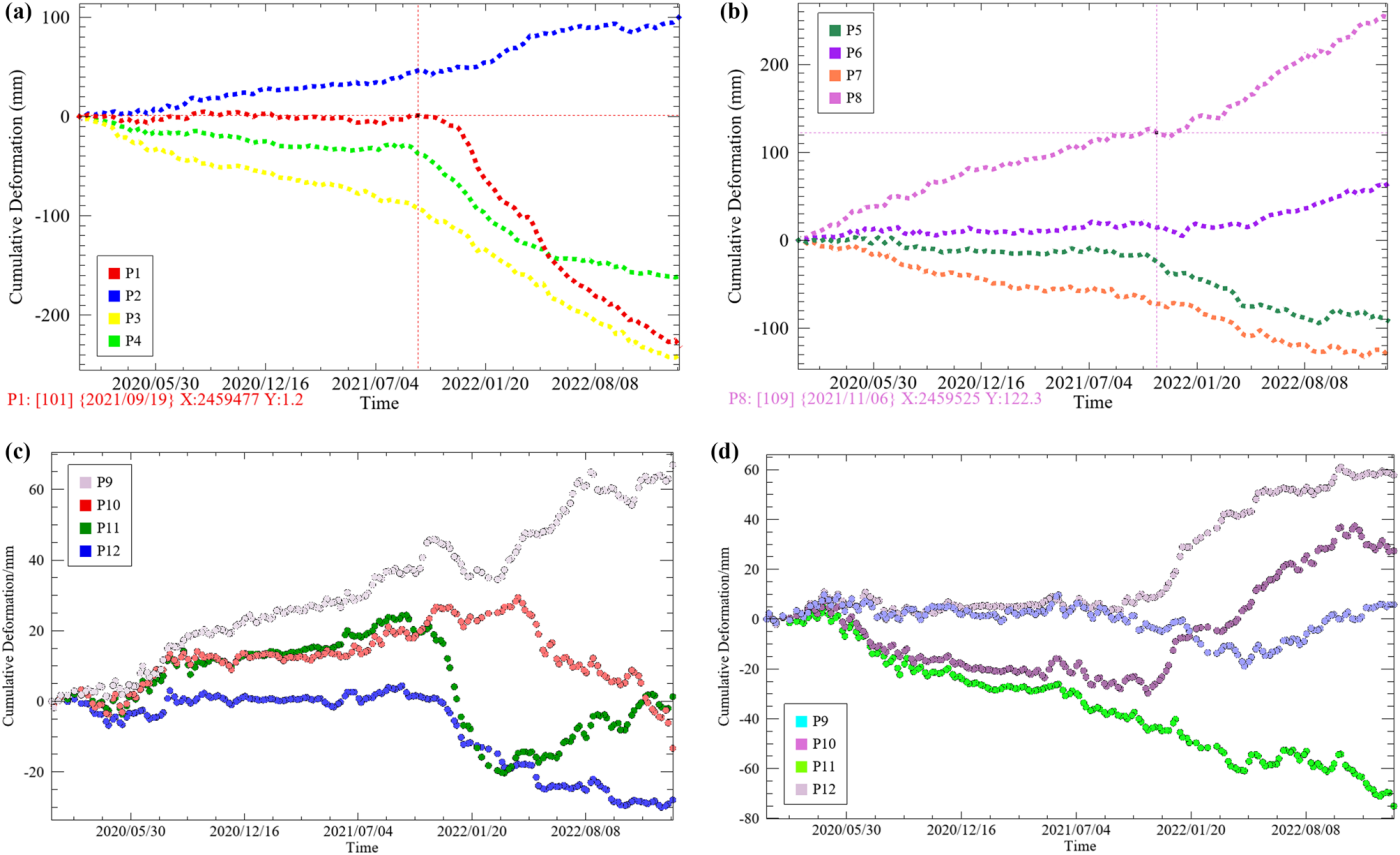
(**a**) Historical deformation curves for feature points P1 to P4, (**b**) historical deformation curves for feature points P5 to P8, (**c**) Historical deformation curves of feature points P9 to P12 in the vertical direction and (**d**) Historical deformation curves of feature points P9 to P12 in the east–west direction.

Joshimath is located in India's Uttarakhand, at the foot of the Himalayas. As an important frontier town in northern India, Joshimath is a unique geographical and topographical stronghold, a cultural center for tourists on pilgrimage to ancient sites, and a strategic route for the Indian army to the north. The town developed on ancient glacial drift deposits and is surrounded by two rivers, the Dhauli Ganga and the Alaknanda (see Fig. 1). The overall elevation of the mountain ranges from 1400 to 2300 m. It is mainly composed of a high density of discontinuous rocks such as quartzite, with frequent features such as faults, fractures, and joints, making the mountain particularly fragile and vulnerable to other factors^27^. In a recent study by Agarwal et al., the town is part of a hotspot that is experiencing rapid climate change and human activity. The extreme climate type has led to frequent flooding disasters in the area and constant river erosion has led to the gradual erosion of the hills on the side of the river valley, the result of this erosion is reflected in the leaning and destruction of pillar structures such as trees, buildings, and electricity poles^28^. According to statistics, the town currently has around 20,000 permanent residents and over 4000 basic buildings. Rapid population growth and urbanization are unscientific and extremely dangerous for a town with steep slopes in the mountains^29^. The impact of human activity is mainly seen in long-term, large-scale infrastructure construction, where inadequate levels of construction and incomplete feasibility analysis at the early stages of construction have resulted in the potential for geological hazards from the works not being detected in time, a most typical example of which is the Tapoban hydroelectric project built on two sections of the river (see Fig. 2). During the study time of 2020–2023, the town was threatened by several severe natural hazards that may have been a major factor in the occurrence of subsidence hazards in the town of Joshiamth. These include the glacial landslide and debris flow event caused by the Nanda Ghunti landslide on 7 February 2021; and three earthquakes of M 4.5 or greater that occurred in the vicinity of the town (see Fig. 3), details of which are given in Table 1.

**Table 1:** Information on earthquakes near Joshimath.

Earthquake center	Magnitude of earthquake	Time	Distance from Joshimath (km)
29 km NE of Bageshwar	M 4.7	2020-2-8	70.1
45 km NW of Joshimath	M 4.5	2021-5-23	45.4
15 km WNW of Pipalkoti	M 4.7	2021-9-11	28.4

## Dataset and methodology

### Dataset

In this paper, Sentinel-1A data were selected as the primary data source for InSAR monitoring, and a total of 178 SLC images covering the study area were acquired for the period January 2020–January 2023, with a revisit period of 12 days. The Sentinel satellite is a geohazard monitoring satellite constructed as part of ESA's Copernicus Earth observation program, which was launched in 2014 and started continuous observation of ground penetrations^30^. The external DEM data used to simulate and remove the topographic phase is essential in the process of InSAR data interpretation^31^. According to Lu^32^ et al., in areas of high topographic relief, the involvement of SRTM1 data allows for better deformation results to be solved, so the SRTM1 with higher accuracy is selected as the reference DEM data with the same resolution. The geoidal height of SRTM1 data is also converted to ellipsoidal height to avoid some errors caused by the geoid. The orbit information is corrected using AUX_POEORB satellite precision orbit data to remove the systematic errors caused by the orbit information. Atmospheric delay errors are corrected or removed using GACOS water vapor data^33,34^, and the basic parameters of the data are shown in Table 2.

**Table 2:** Basic parameter information of experimental data.

Dataset	Parameters	Information	
Sentinel-1A	Path	Ascending	Descending
		129	63
	The angle of incidence (°)	43.76	35.76
	Polarization	VV	VV
	Number	91	87
SRTM1	Elevation datum	WGS84/EGM96	
	Resolution (m)	30	
	Elevation accuracy (m)	16	
AUX_POEORB	Positioning accuracy (cm)	5	

The EGM96 model is a high-precision geodetic level model calculated and published in the USA for global application.

### MSBAS-InSAR processing

The SBAS-InSAR technique was first proposed by Berardino et al.^35–37^ and the basic idea is to obtain the same Multiple SAR images of a region are selected and pixels that meet certain conditions on these images are selected for time series analysis. Compared with other time-series InSAR techniques, the SBAS-InSAR technique is unique in that it requires the processing of multiple interferometric pairs, and the temporal and spatial baselines of the interferometric pairs need to be kept within a certain threshold range at the same time, and the interferometric pairs are subjected to multi-visualization in phase space, which reduces the spatial resolution of the image but also ensures the maximum coherence of the interferometric phase.

Assuming that there are N + 1 SAR images covering the same study area and arranged in temporal order t0, t1…tn, a common master image is automatically selected and aligned with the other slave images one by one. Based on the combination of the interferometric conditions, N + 1 SAR images can be combined into M interferograms, where the size of M will satisfy the following conditions:

1 $$\begin{array}{c}\frac{N+1}{2}\le M\le \frac{N\left(N+1\right)}{2}\end{array}$$

If the jth differential interferogram is obtained from the differential interference of the two phases of SAR images acquired from the moments ${t}_{A}$ and ${t}_{B}({t}_{B}>{t}_{A})$, then the following relationship exists between the interferometric phase of its pixels and the azimuthal coordinates x and distance-oriented coordinates r. The relationship equation is:

2 $$\begin{array}{c}\delta {\phi }_{j}\left(x,r\right)=\varphi \left({t}_{B},x,r\right)-\varphi \left({t}_{A},x,r\right)\approx \frac{4\pi }{\lambda }\left[d\left({t}_{B},x,r\right)-d\left({t}_{A},x,r\right)\right]\end{array}$$

where $j\in (1\sim M)$; λ is the central wavelength of the radar signal; $d({t}_{B},x,r)$ and $d({t}_{A},x,r)$ are the cumulative deformation variables of the radar line of sight direction relative to $d\left({t}_{0},x,r\right)=0$ at the moments ${t}_{B}$ and ${t}_{A}$, respectively.

To express the time series of surface deformation as a mathematical expression with physical meaning, the average phase velocity in Eq. (2) is expressed as the product of the average phase velocity and time over the two observed times obtained, then the average phase velocity can be expressed as:

3 $$\begin{array}{c}{v}_{j}=\frac{{\varphi }_{j}-{\varphi }_{j-1}}{{t}_{j}-{t}_{j-1}}\end{array}$$

The phase value of the jth amplitude interferogram is expressed as the integral of the average rate over each period in the primary and secondary images and is expressed as:

4 $$\begin{array}{c}\sum_{k={t}_{A},j+1}^{{t}_{B},j}\left({t}_{k},{t}_{k-1}\right){v}_{k}=\delta {\varphi }_{j}\end{array}$$

The differential interferograms are combined and written in matrix form as:

5 $$\begin{array}{c}Bv=\delta \varphi \end{array}$$

where ***B*** denotes the coefficient matrix of *M* × *N*. Then ${v}^{T}$ can be expressed as:

6 $$\begin{array}{c}{v}^{T}=\left[{V}_{1}=\frac{{\varphi }_{1}}{{t}_{1}-{t}_{0}},\dots ,{v}_{N}=\frac{{\varphi }_{N}-{\varphi }_{N-1}}{{t}_{N}-{t}_{N-1}}\right]\end{array}$$

When *M* ≥ N, i.e., the coefficient matrix is full rank, then the estimate of φ can be solved using least squares:

7 $$\begin{array}{c}\widehat{\varphi }={\left({B}^{T}B\right)}^{-1}{B}^{T}\delta \varphi \end{array}$$

When *M* ≤ N, i.e., the coefficient matrix ***B*** is prone to rank deficit, and the generalized inverse matrix of ***B*** is calculated according to the singular value decomposition (SVD) method to obtain the minimum parametric solution for the deformation rate ***v***. The final method is used for each period and then accumulates to obtain the cumulative deformation rate over the entire time horizon.

The LOS direction deformation results of the surface can be obtained by the above method, but to more accurately determine and simulate the real surface deformation values, a binary system of equations was further developed based on a mathematical model of multi-source data fusion^38^ for the InSASR observations and the parameters to be solved for the deformation rates and temporal deformation variables of the lift trackSAR data in the vertical and east–west directions during the overlapping periods.

Since most SAR satellites currently fly in near north–south orbits, resulting in insensitivity to azimuthal deformation monitoring^39^, north–south deformation is ignored. That is, a system of equations can be formulated as:

8 $$\begin{array}{c}\left\{\begin{array}{c}{D}^{Ascending}={D}_{v}\cdot \mathrm{cos}{\theta }_{1}-{D}_{e}\cdot \mathrm{cos}{\varphi }_{1}\mathrm{sin}{\theta }_{1}\\ {D}^{descending}={D}_{v}\cdot \mathrm{cos}{\theta }_{2}-{D}_{e}\cdot \mathrm{cos}{\varphi }_{2}\mathrm{sin}{\theta }_{2}\end{array}\right.\end{array}$$

The matrix form is as follows:

9 $$\begin{array}{c}{D}^{0}={B}^{0}\cdot {D}_{ev}\end{array}$$

where $${D}^{0}={\left[{D}^{Ascending} {D}^{descending} \right]}^{T}$$;$${D}_{ev}={\left[{D}_{v} {D}_{e} \right]}^{T}$$;$${B}^{0}=\left[\begin{array}{cc}\mathrm{cos}{\theta }_{1}& -\mathrm{cos}{\varphi }_{1}\mathrm{sin}{\theta }_{1}\\ \mathrm{cos}{\theta }_{2}& -\mathrm{cos}{\varphi }_{2}\mathrm{sin}{\theta }_{2}\end{array}\right]$$.

To avoid the pathology of the observation equation, the design matrix needs to be regularized. The observation equation is expressed as:

10 $$\begin{array}{c}\left(\begin{array}{c}A\\ \xi I\end{array}\right)V=\left(\begin{array}{c}{D}_{0}\\ 0\end{array}\right)\end{array}$$

where $\mathrm{A}=(-\mathrm{cos\varphi sin\theta }\Delta \mathrm{t},\mathrm{cos\theta }\Delta \mathrm{t})$ is the design matrix: $\Delta \mathrm{t}$ is the adjacent SAR image time interval; $\xi$ is the regularization factor; *I* is the unit matrix; ${D}_{0}$ is the deformation phase matrix; $D=({D}_{e},{D}_{v}{)}^{T}$, ${D}_{e},{D}_{v}$ are the parameters to be solved for and their valuations can be expressed as:

11 $$\widehat{D}=({A}^{T}A+\xi I{)}^{-1}{A}^{T}D$$

**Figure 4:**
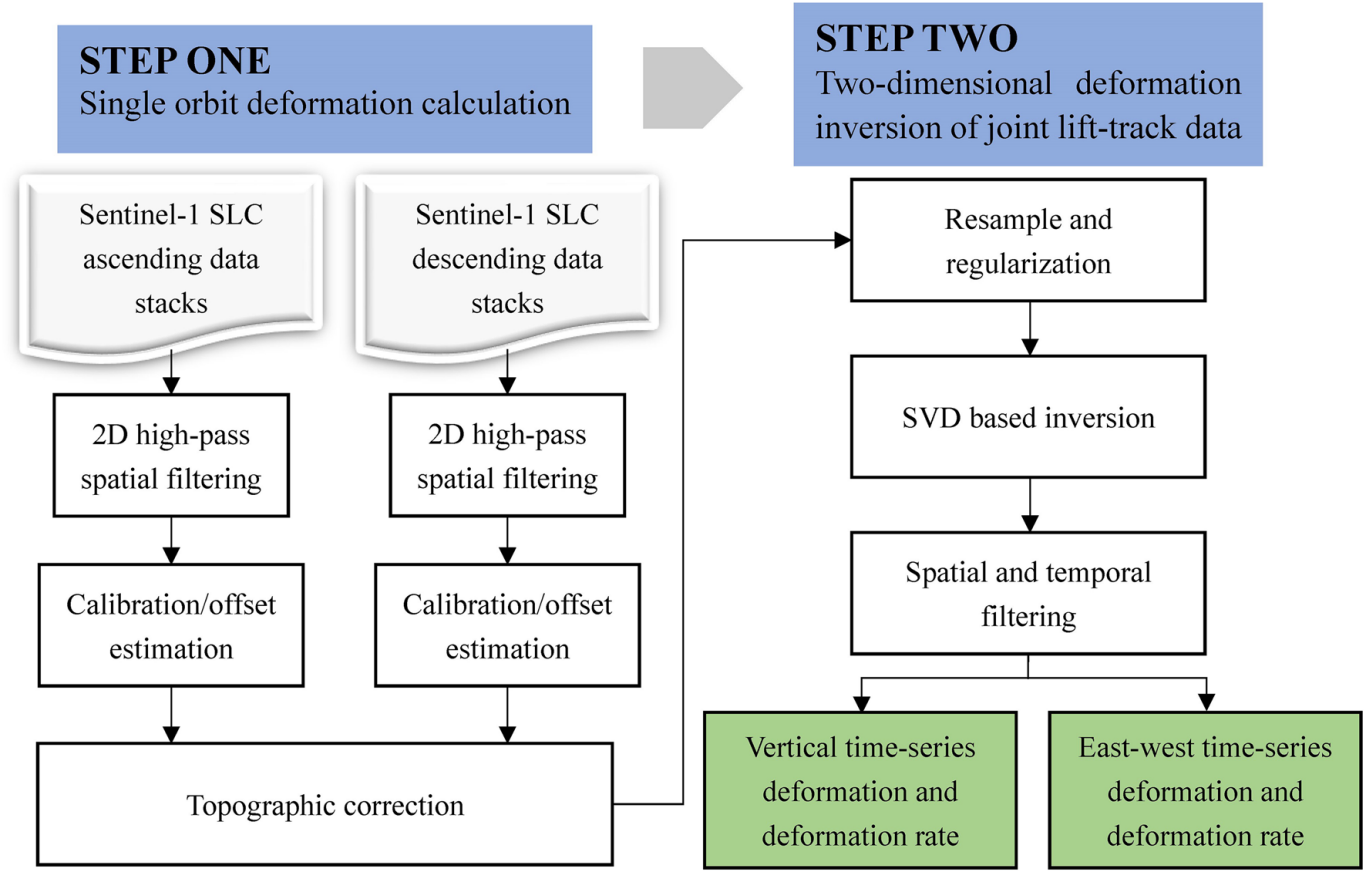
The flow chart of multidimensional deformation monitoring using MSBAS-InSAR technology.

Finally, the deformation rates between adjacent SAR acquisition moments are integrated into the time domain to obtain the east–west and vertical deformation time series of the ground surface:

12 $$d_{E,i} = \sum\limits_{i = 1}^{n} {D_{E,i} \Delta t_{i} ,} \;\;\;\;d_{U,i} = \sum\limits_{i = 1}^{n} {D_{U,i} \Delta t_{i} }$$

where d is the cumulative deformation time series; n is the total number of period coincidences of different orbital images. This is the principle flow of the MSBAS-InSAR technique, and the processing flow chart is shown in Fig. 4.

## Results

**Figure 5:**
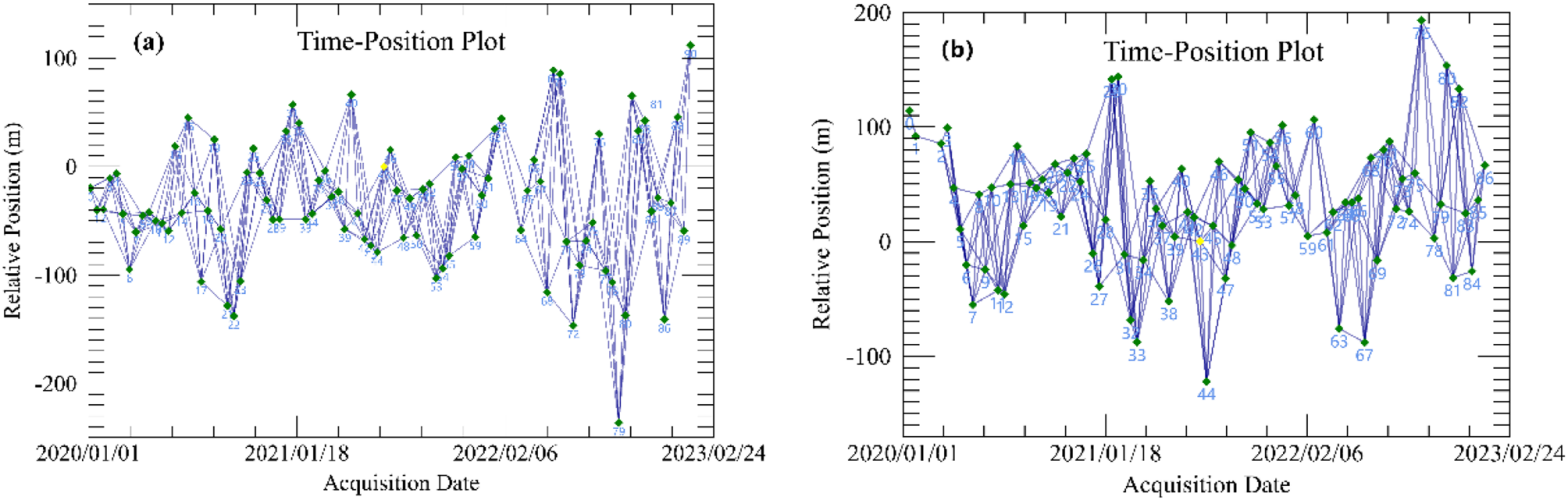
(**a**) Baseline connection diagram for ascending and (**b**) baseline connection diagram for descending.

**Figure 6:**
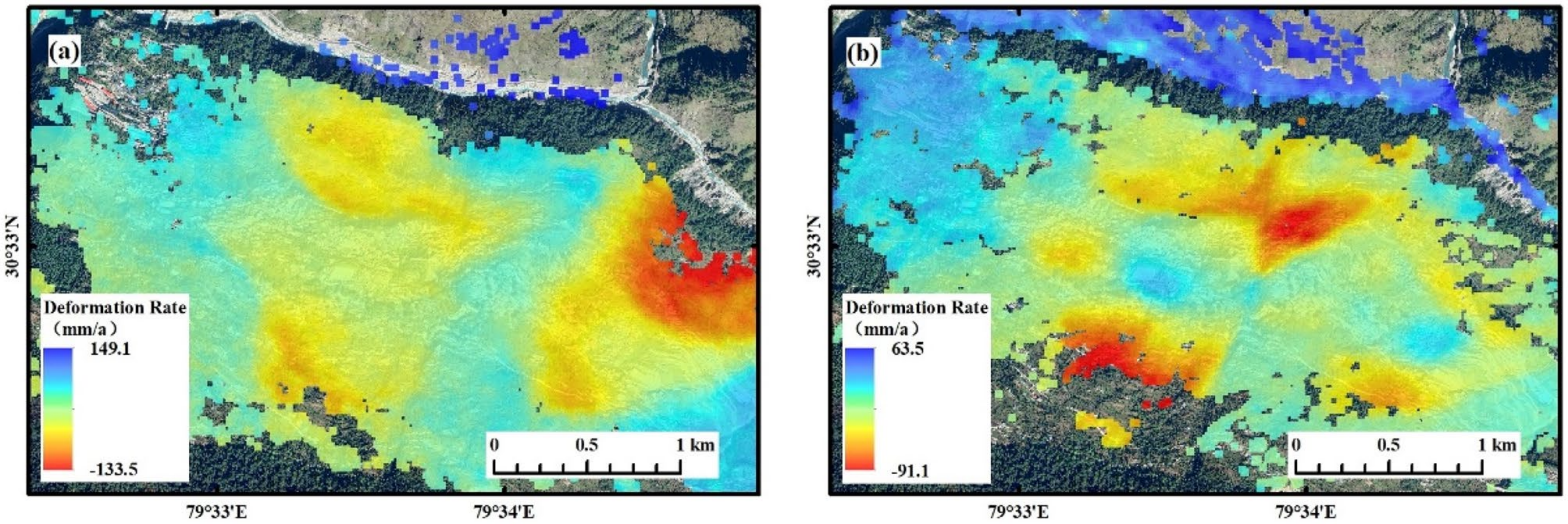
(**a**) Annual deformation rate in the LOS direction of the ascending and (**b**) annual deformation rate in the LOS direction of the descending.

**Figure 7:**
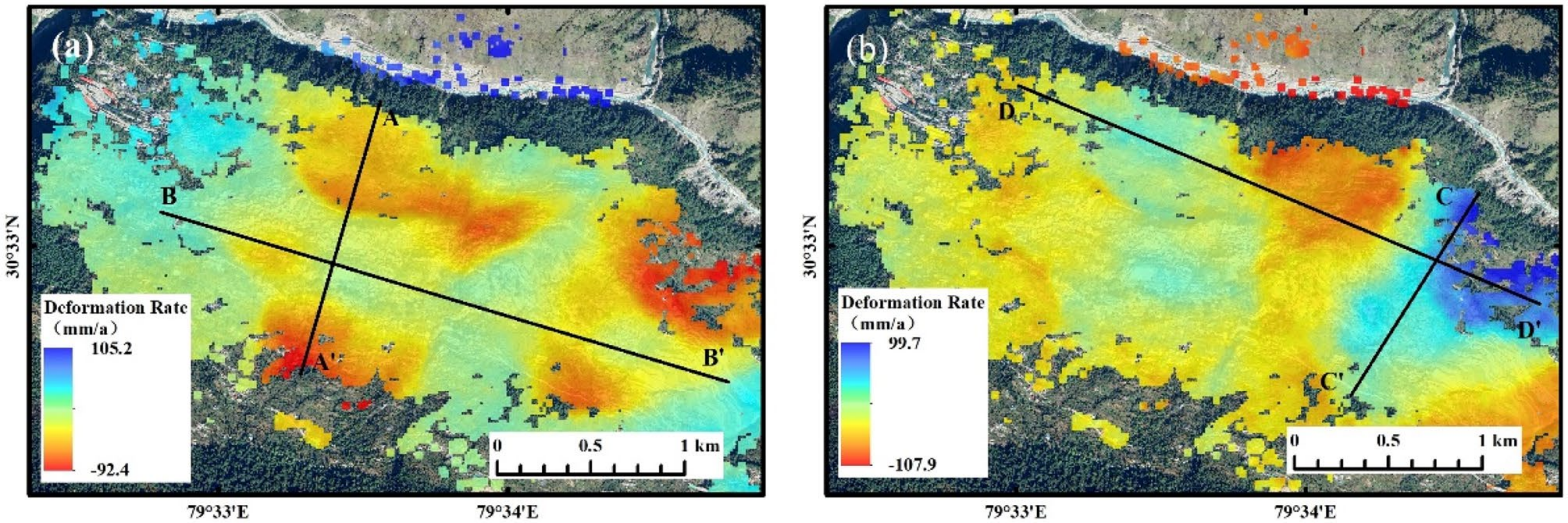
(**a**) Annual deformation rate in the vertical direction and (**b**) annual deformation rate in the east–west direction.

Separate SBAS-InSAR processing of ascending and descending SAR images, and the maximum time baseline was set to 48 days and the maximum spatial baseline to 10% of the critical baseline to obtain a higher coherent interferometric pair. The spatial baseline connectivity of the pairs is shown in Fig. 5, where the X-axis represents time and the Y-axis represents the baseline distance between the master image and each slave image. The yellow and green points shown represent the distribution of the master and slave images in the time series, where the SAR image of 27 June 2021 was selected as the master image for the ascending track. The main image of the descending orbit was taken on 17 July 2021, finally, a total of 347 sets of up-track and 325 sets of down-track interference pairs were generated. In addition, the azimuth looks and range looks were set to 4 and 1, and the grid size for suggested looks was set to 15 m. A Goldstein filter was used to remove the noise phase^40^, and the filtered interferogram was phase-disentangled using the minimum cost flow (MCF) method^41^. Finally, the SAR plane coordinates were projected to the WGS-84 coordinate system by geocoding, and a product output coherence threshold of 0.3 was set to obtain the InSAR deformation field in the LOS direction (see Fig. 6). A joint-weighted least squares inversion was performed based on the SBAS-InSAR deformation results of the lift track to obtain the deformation rates and time-series deformation characteristics of the vertical and horizontal east–west directions of the Joshimath surface (see Fig. 7).

As can be seen from Fig. 6, the annual deformation rate of the surface observed in the ascending orbit ranges from -133.5 to 149.1 mm/a, while the deformation rate in the descending orbit ranges from − 91.1 to 63.5 mm/a. There are some differences in the spatial distribution characteristics of the ascending and descending InSAR deformation fields, and even opposite deformation trends exist in some areas, which are related to the different directions and incidence angles of SAR imaging^42^. At the root of this is the characteristic of SAR right-view imaging, where the projection of the horizontal movement of the surface in the direction of radar LOS shows the opposite movement characteristics when the radar observes the ground in two orbits in opposite directions^43^. Thus, the difference in the spatial distribution of the InSAR deformation field of the lift rail suggests that there is both vertical and horizontal movement of surface deformation in the town of Joshimath and that the degree of deformation is greater in the horizontal direction than in the vertical direction. That is, if only single-orbit InSAR monitoring is carried out, erroneous deformation results may be produced due to the singularity of the observations.

Figure 7 shows that there is significant vertical and horizontal east–west deformation in the town of Joshimath, where positive deformation rates indicate surface uplift or eastward movement, and negative values indicate surface subsidence or westward movement. The main deformation areas are in the east-central part of the town, where the surface subsidence is irregularly distributed and the horizontal movement is funnel-shaped, with the largest deformation rates occurring in the northeastern part of the town. The remote sensing images show that this is the headwaters of the confluence of the Dhauli Ganga with two other rivers and that the riverbed has been deformed by the swift flow of the river, exposing a large amount of grey rock. Therefore, the impact of extreme rainfall, flooding, and mudslides may be a factor in the high rate of deformation here. The Badrinath Road on the outskirts of the town is also characterized by significant deformation, and as an important transport road, the collapse of the road surface would have a serious impact on people's daily lives.

### Spatial evolutionary characterization

**Figure 8:**
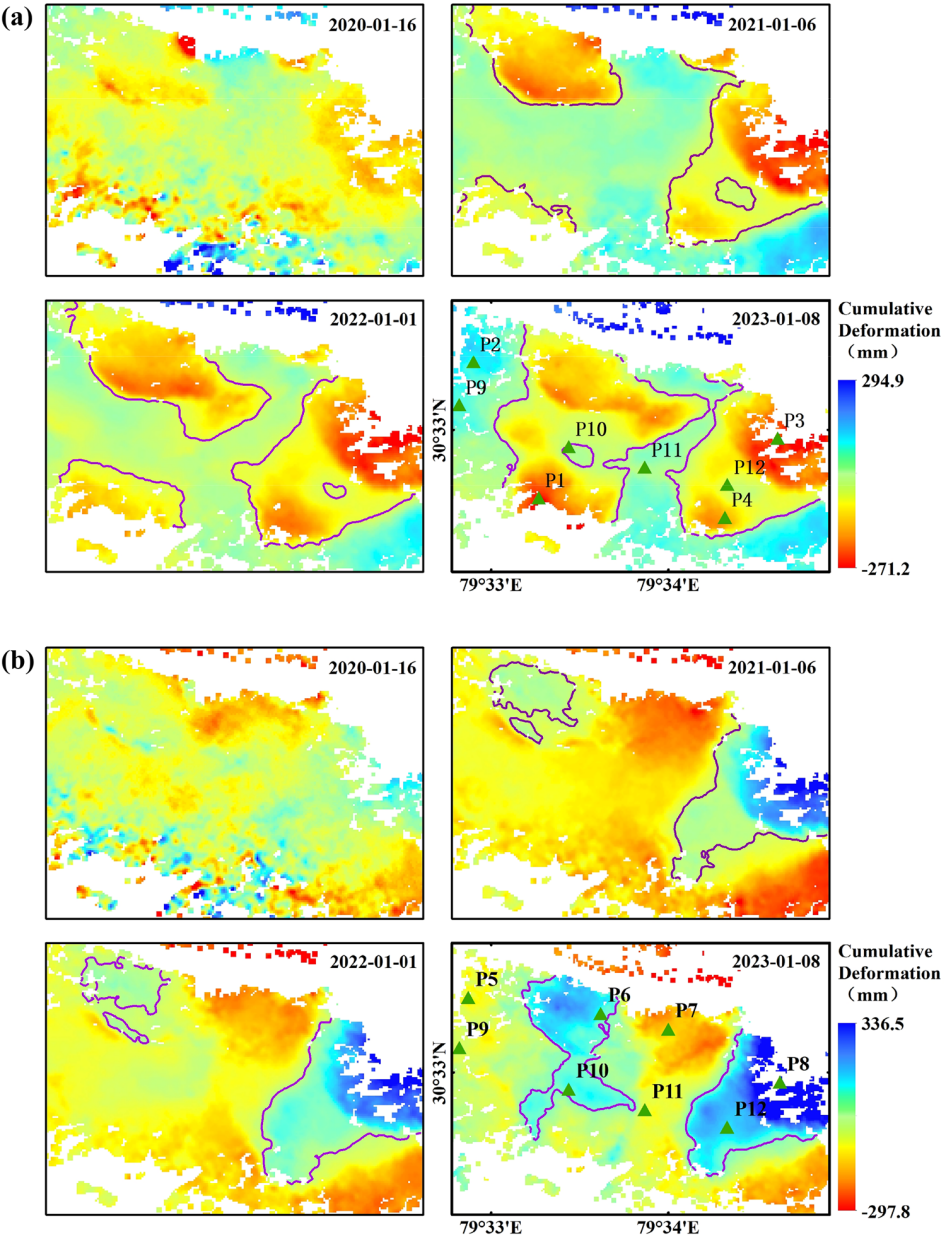
(**a**) Cumulative deformation sequence in the vertical direction and (**b**) cumulative deformation sequence in the horizontal east–west direction.

**Figure 9:**
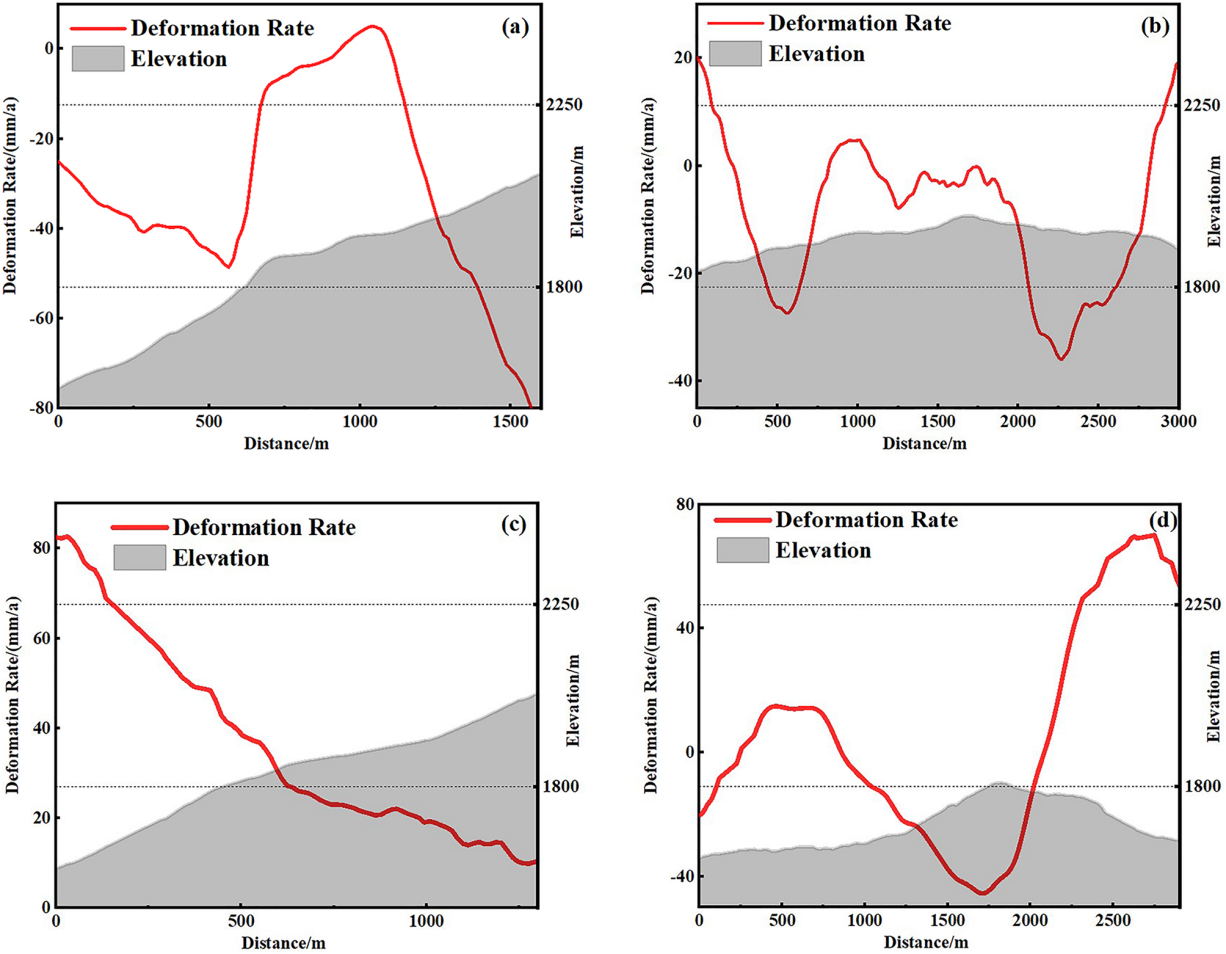
Typical deformation rate profiles: (**a**) A–A′ section line, (**b**) B–B′ section line, (**c**) C–C′ section line, and (**d**) D–D′ section line.

As can be seen in Fig. 8, both the extent and size of surface deformation gradually increased over time. The extent of deformation of the surface in the eastern part of the town basically took shape 1 year after beginning monitoring, and the surface began to accumulate deformation within a certain range, while the extent of deformation in the central and western parts of the town continued to expand during the monitoring period, especially after 2022, and the rate and extent of deformation of the surface increased significantly and continued to accelerate after 2023. By vector statistics, the area of ground subsidence is about 3.35 km^2^, accounting for 36% of the total area of the town of 9.22 km^2^; the area of eastward movement is about 2.31 km^2^, accounting for 25% of the total area of the town. The spatial and temporal evolution of the deformation shows that the distribution of ground subsidence and horizontal movement is based on the lower part of the hill towards the hill, which correlates with the topographic changes of the hill on which the town is located. Therefore four profile lines in two directions (A–A′, B–B′; C–C′, D–D′)as shown in Fig. 6 were plotted to extract information on the deformation rate and topography at the location of the profile lines, as shown in Fig. 9. It can be seen that ground subsidence with deformation rates ≥ 20 mm/a mainly occurs in areas below 1800 m and above 2000 m above sea level, and surface subsidence in the elevation range of 1800–2000 m is relatively stable. The rate of horizontal ground movement gradually decreases as the terrain rises, and on the relatively gentle east–west terrain, the ground moves repeatedly in a west–east direction in sequence and at a progressively increasing rate. In summary, excluding the effects of natural disasters and anthropogenic activities, the surface deformation of Joshimath town is related to the topography of the mountain, with the rate of surface subsidence relatively rapid when the topography is high or low, compared to the rate of horizontal movement which decreases as the topography rises.

### Time evolutionary characterization

To further quantify the evolution trend of surface deformation over time, the historical deformation information (see Fig. 3) was extracted from twelve feature points (P1–P12) as shown in Fig. 2, and their deformation characteristic was analyzed to derive the deformation trend of Joshiamth urban area from local to overall. P1 to P3 is located in the main subsidence zone, P4 in the main uplift zone, P5 to P8 in the horizontal movement deformation zone, and P9 to P12 is located on the east–west median of the town. In Fig. 8, different feature points all show non-linear deformation trends at different rates, the surface deformation zone at P4 continues to lift at a relatively constant rate, with a cumulative uplift of 113 mm as of 8 January 2023. The surface deformation in the three typical subsidence areas goes through two phases, with almost no ground subsidence occurring at point P1 in the first phase and relatively flat subsidence rates at points P3 and P4. In the second phase, after 19 September 2021, the subsidence rates at the three characteristic points show a relatively large increase. It is only after 20 January 2022 that the cumulative subsidence at point P4 stabilizes, while points P1 and P3 continue to settle at the same rate and do not slow down after 8 January 2023. Similarly, feature points P5 to P8 continue to move at a relatively steady rate in their respective directions until a point in time in the second half of 2021 when the rates of movement at each feature begin to show an increasing trend. But in contrast, the horizontal movement rate increases less than the subsidence rate, and the point in time at which the rate increases slightly lags behind the latter, indicating that horizontal movement is less affected and vertical movement is more susceptible to stimulation and influence from the external environment due in part to gravity. Over time, after August 2022, the movement of the P5 and P7 feature points gradually stabilizes, with the final cumulative settlement remaining at around 100 mm. This indicates that the horizontally westward moving deformation zone in which P5 and P7 are located has largely stabilized before the study cut-off date, while the horizontal eastward movement of the ground surface has still not ceased. The deformation trends of the feature points from P9 to P12 in the vertical and east–west directions are consistent with the other feature points, but the degree of deformation is smaller, indicating that the urban area of the town is less affected than the suburban area.In summary, the area of maximum cumulative deformation is located in the northeastern part of the town, and the maximum cumulative surface settlement is 271.2 mm, with a cumulative horizontal movement of 336.5 mm to the east, the trend of deformation has not slowed down and the cumulative deformation variables continue to increase.

## Discussion

**Figure 10:**
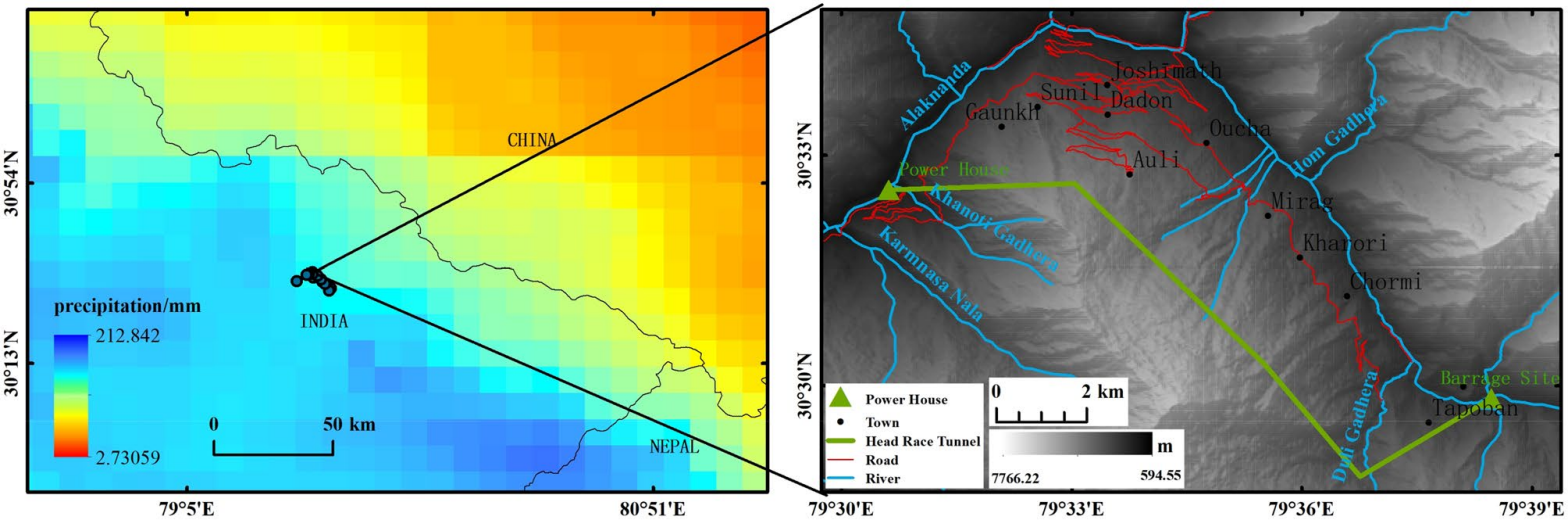
Average monthly precipitation in the area around Joshimath and Tapoban hydroelectric project plan.

**Figure 11:**
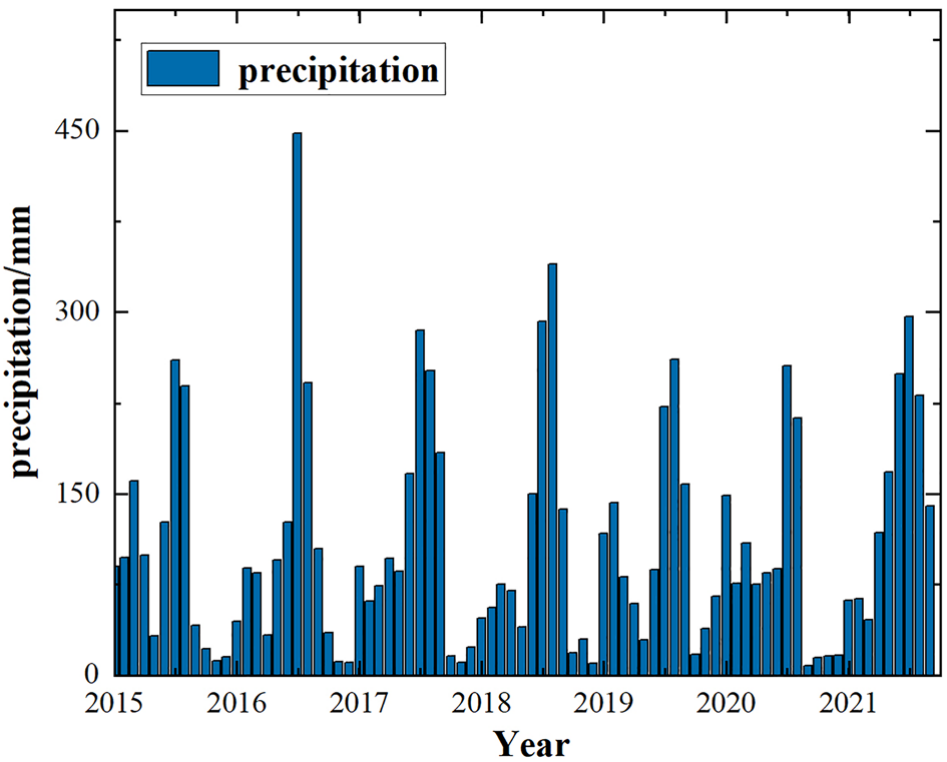
Precipitation changes in Joshimath from 2015 to 2022.

The Tapoban Hydroelectric Project aims to create a diversion tunnel in the mountainous area where the town of Joshimath is located^44^ And build a water storage dam near the town of Tapoban to convert the gravitational potential energy of the high water level upstream into kinetic energy through a tunnel to drive the power station at the Alaknanda River downstream to generate electricity. However, the large tunneling must have damaged the geological structure of the mountain and the groundwater system, resulting in the stability of the town's foundations being reduced in the mountains. And with the natural topographic barrier causing the town to receive around 109 mm/month of rainfall between 2015 and 2022^45^ (See Figs. 10 and 11), the large amount of extreme precipitation leaching into the town's aquifers from high altitudes via rivers and roads could further exacerbate the damage to the town's foundations.

In the landslide that occurred in February 2021^46^, the massive amount of rock and glacier fall from the mountain peaks to the valley floor generated so much energy that the landslide transitioned into a mudslide. The raging mud flowed through the river into the Dhauli Ganga and continued to advance to Alaknanda causing damage to the valley and inflow tunnel of the mountain where Joshimath is located. Not only does this pose a serious threat to human production and natural resources, but it could also become a new destabilizing factor in the destruction of the town's foundation structure. However, judging from the trend of the historical deformation curve in Fig. 9 on and around February 7, 2021, neither the construction of the hydroelectric project nor the effects of flooding hazards such as extreme rainfall and mudslides are decisive factors in the occurrence of the eventual subsidence hazard in the town of Joshiamth.

**Figure 12:**
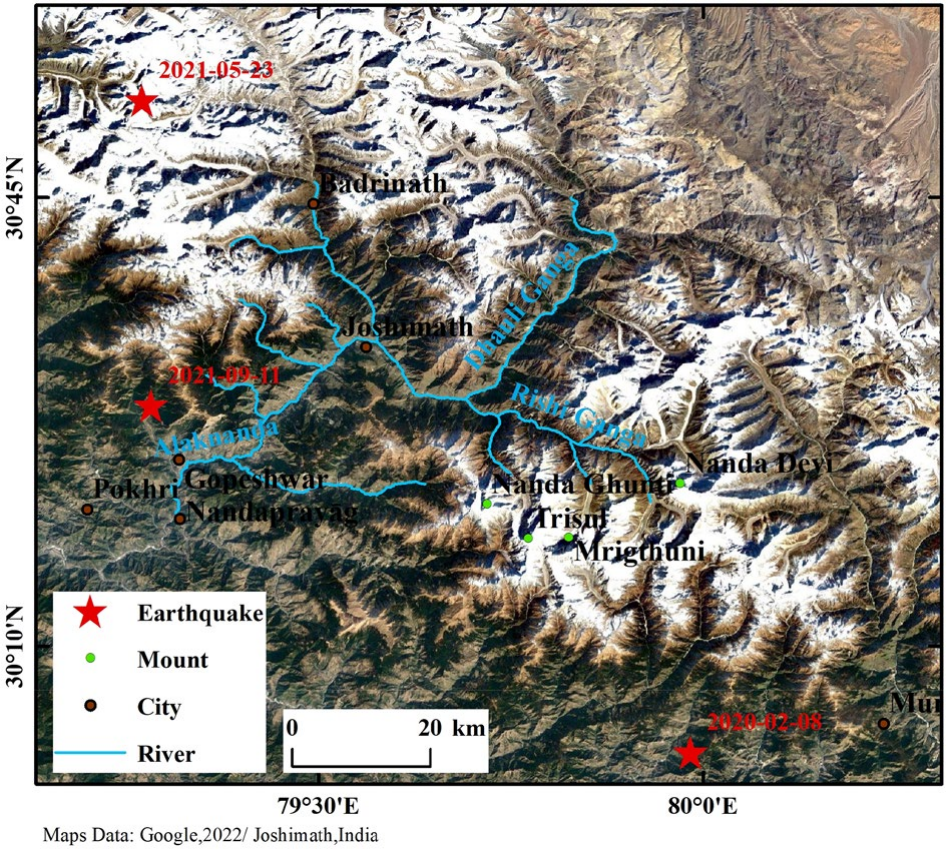
Location of reported earthquakes. Source: USGS.

The timing and location of the seismic hazards in Table 1 and Fig. 12 show that the nearest epicenter is only 28.4 km from Joshiamth, in the hills to the southwest of Joshimath. The timing of the earthquake, on 11 September 2021, coincides highly with the point at which the town's subsidence rate changed. It is therefore tentatively concluded that the earthquake was a decisive factor in the onset of the subsidence hazard in Joshimath and that the rate of surface deformation was significantly increased by the earthquake, leading to an earlier onset of the geological subsidence hazard in the town. However, because of the low magnitude of the earthquake, the spatial distribution of the seismic fault could not be monitored by InSAR and the true magnitude and extent of the deformation could not be determined, so further field studies are needed to summarise the conclusions of this paper.

Joshimath's unprecedented subsidence disaster has a long history, due to urban development and the destruction of natural water channels, due to the constant erosion of the mountain by rivers, and due to human activity at all levels breaking down the underlying structure of the mountain surface. After the first obvious signs of surface movement were ignored or disregarded, various instability factors reached maturity over time, and eventually a more serious geological subsidence disaster occurred as a result of a natural disaster. More importantly, the movement of landslide complexes is highly complex and it is unlikely that a landslide will achieve permanent stability in its current physical state, even if it exhibits a relatively stable state in the short term after the intervention of anthropogenic activities has been excluded. The current crisis may lift at some point thereafter, but potential future crises may persist. Therefore, foreseeable geological hazards such as these, which we cannot afford to repeat Lessons should be learned and lessons learned, and the task of disaster prevention and mitigation should be adjusted to a manageable level while conducting adequate geological studies and disaster prevention and control.

## Conclusions

In this paper, the MSBAS-InSAR technique was used to monitor and process the surface deformation of Joshimath town over the past three years based on Sentinel-1A data, and the deformation rates and time series characteristics of the town's vertical and horizontal east–west directions were obtained. The spatial distribution and spatial–temporal evolution of the surface deformation were analyzed in detail, and the mechanism and trend of the surface deformation were summarised. And the following conclusions are drawn.

1. There is significant vertical and horizontal east–west deformation in the town of Joshimath, with annual deformation rates ranging from − 92.4 to 105.2 mm/a in the vertical direction and − 107.9 to 99.7 mm/a in the horizontal east–west direction. The area of ground subsidence is approximately 3.35 km^2^, accounting for 36% of the total area of the town of 9.22 km^2^; The area of greatest cumulative deformation is located in the northeastern part of the town, with a maximum cumulative ground settlement of 271.2 m and a cumulative horizontal movement of 336.5 m to the east.
2. The spatial evolution of surface deformation in Joshiamth is closely related to the topography of the mountain. The distribution of ground subsidence and horizontal movement is based on the base of the mountain and progresses up the mountain, showing an expansion from the bottom to the top. The rate of horizontal movement decreases with increasing elevation, while the rate of subsidence evolves in a non-linear trend of rising, then falling, then rising again with increasing topography.
3. The temporal evolution trend is broadly divided into two phases: the first phase has a relatively flat rate of surface deformation, and the second phase after October 2021 has a significant increase in the rate of deformation. But in contrast, the increase in the rate of horizontal movement is less than the rate of subsidence, which indicates that the horizontal movement is less affected and the vertical movement is more easily stimulated and influenced by the external environment due to gravity and other factors.
4. The preliminary judgment is that anthropogenic activities such as engineering construction and flooding disasters such as extreme rainfall are among the destabilizing factors that undermine the foundation structure of the town and are among the causal factors for the continuous deformation of the ground surface. However, the dramatic change in the rate of surface deformation and the early onset of the geological subsidence hazard was triggered by the 4.7 magnitude earthquake that struck near the town on 11 September 2021.

## Data Availability

The data presented in the study are available on request from the first and corresponding author. The data are not publicly available due to the thesis that is being prepared from these data.
